# Enhancing genetic variability in *Trigonella* species through sodium azide induction: morpho-physiological and chromosomal amelioration

**DOI:** 10.3389/fgene.2024.1378368

**Published:** 2024-05-09

**Authors:** Neha Naaz, Sana Choudhary, Nazarul Hasan, Nidhi Sharma, Khadiga Alharbi, Diaa Abd El Moneim

**Affiliations:** ^1^ Cytogenetics and Plant Breeding Section, Department of Botany, Aligarh Muslim University, Aligarh, India; ^2^ Department of Biology, College of Science, Princess Nourah bint Abdulrahman University, Riyadh, Saudi Arabia; ^3^ Department of Plant Production (Genetic Branch), Faculty of Environmental Agricultural Sciences, Arish University, El-Arish, Egypt

**Keywords:** plant breeding, crop genetic improvement, fenugreek genotype, genetic diversity, chromosomal alterations

## Abstract

Plant breeding, aimed at enhancing desired traits, depends on genetic diversity. Mutation breeding is a powerful method of rapidly expanding genetic diversity, facilitating crop improvement, and ensuring food security. In a recent study, researchers evaluated the genetic variability of *Trigonella* species using different doses of sodium azide (SA) (0.2%, 0.4%, 0.6%, 0.8%, and 1.0%) through morphological, physiological, and cytogenetic studies. Morphological variations were observed in cotyledonary leaves, vegetative leaves, and overall plant growth and habit. Several quantitative parameters, such as plant height, fertile branches per plant, pods per plant (or clusters), seeds per pod, and seed yield, increased when treated with 0.2% and 0.4% SA compared to the control. Furthermore, the total chlorophyll content and carotenoids increased in the sample treated with 0.2% SA over the control but decreased with higher concentrations. Scanning electron microscopy revealed that stomatal aperture and seed dimensions increased at lower concentrations of sodium azide treatment. The study found a positive correlation between the different parameters studied in the *Trigonella* species, as indicated by high r-values. Based on their findings, it was concluded that the genotype of fenugreek can be improved by using 0.2% and 0.4% concentrations of sodium azide. However, the evaluation of observed variants in successive generations is a critical and necessary process to validate their potential as keystones for crop genetic improvements.

## Introduction

Mutation breeding is highly regarded by plant breeders as an effective method of enhancing crop productivity and achieving sustainable crop production. It introduces diversity and variation into crops, leading to the development of varieties with higher yields, disease resistance, and improved tolerance to climate change. This approach identifies important regulatory genes, enabling the alteration of specific traits in well-adapted varieties (Agrawal and Kumar, 2021; Anter, 2023). The primary methods for inducing plant mutations are irradiation and chemical mutagen treatment, with chemical mutagens offering the advantage of generating mutant populations with high mutation densities (Szarejko et al., 2017). These mutagens cause physiological damage, macro- and micro-mutations in genes, and chromosomal abnormalities in the population, often inducing point mutations. Sodium azide (SA) is an example of a chemical mutagen that has shown promising results in enhancing agronomic traits through enzymatic conversion to L-azidoalanine (Owais and Kleinhofs, 1988). SA-induced mutagenesis has been observed in various crop species, including fenugreek, wheat, maize, rice, and rapeseed (Siddiqui et al., 2007; Srivastava et al., 2011; Eze and Dambo, 2015; Herwibawa and Kusmiyati, 2017; Hussain et al., 2017).

Medicinal and aromatic crops, particularly species of *Trigonella*, hold significant importance in the long-term economic development of many countries. *Trigonella*, consisting of over 280 species within the Fabaceae family, thrives in regions such as the Mediterranean, northern India, western Asia, and Africa. Notably, *T. foenum-graecum* (var. Pusa early bunching (PEB)) and *T. corniculata* (Pusa kasuri) are popular spice and leafy vegetable crops, with Rajasthan, India, being a major global producer of fenugreek (Babaleshwar and Shetty, 2017). *Trigonella foenum-graecum* is a fast-growing annual herb with upright stems, trifoliate obovate leaves, and white to pale-yellow flowers that develop into straight pods containing large-sized, non-scented, golden-yellowish seeds (Kavina et al., 2020). The Pusa early bunching (PEB) variety was the best for more herbage parameters resistant to downy mildew and rots. It matures in 125 days from seed to seed. On the other hand, *Trigonella corniculata* is an annual, slow-growing, bushy herb with rosette-like vegetative growth, producing bright orange to yellow flowers and sickle-shaped pods with small-sized and scented seeds (Yadav et al., 2021). Kasuri methi, also known as “Champa methi” or “Marwari methi,” is cultivated as a semi-arid crop during the rabi (spring) season and is recognized for its tolerance to drought and frost, requiring cool and dry weather for maturity (Saxena et al., 2017). The Pusa kasuri variety is a small-seeded type, mainly cultivated for leaf purposes and not for seeds. It is a late flowering variety with rosette-type leaves, and five to seven cuttings may be taken. It is a heavy yielder of green leaves with a special fragrance. Fenugreek seeds and leaves are widely utilized for culinary purposes, enhancing the flavor, color, and texture of food, as well as for medicinal applications (Wani and Kumar, 2018). Fenugreek leaves are particularly rich in essential vitamins and nutrients (Tayade et al., 2021), while fenugreek seeds contain valuable phytochemicals such as steroids, carbohydrates, proteins, flavonoids, amino acids, and volatile oils (Mehrafarin et al., 2010). The seeds are composed of carbohydrates, proteins, fats, and dietary fiber, making them highly nutritious. Fenugreek demonstrates diverse pharmacological properties, including antidiabetic, antioxidant, anti-fertility, antimicrobial, anticarcinogenic, anti-inflammatory, and immunomodulatory effects, among others (Yadav and Baquer, 2014; Reddy et al., 2019). These properties contribute to the widespread use of fenugreek in the food and pharmaceutical industries.

The research aims to investigate the mutagenic effect of sodium azide on two *Trigonella* species, focusing on morphological, physiological, and cytological studies. The findings from this study will contribute to the genetic enhancement of *Trigonella* species, making them more valuable for medicinal, aromatic, and agricultural purposes. By expanding the genetic diversity of these crops, plant breeders can select and cultivate improved genotypes that have the potential to address challenges in agriculture and contribute to sustainable crop production in the long term. Responsible and well-monitored breeding practices can harness the potential of induced variability to create more sustainable and resilient fenugreek crops for the future.

## Materials and methods

Seeds of *T*. *foenum-graecum* and *T*. *corniculata* were obtained from the Indian Agricultural Research Institute (IARI), New Delhi. The research was conducted with proper permissions from the Department of Botany at Aligarh Muslim University (AMU), Aligarh, and consent from IARI, New Delhi. The seeds were surface sterilized with 5% sodium hypochlorite for 10 min and rinsed with distilled water. Then, the germplasm was pre-soaked in distilled water for 12 h and mutagenized with five concentrations of SA for 9 h, with untreated seeds as a control (Naaz et al., 2023). Solutions of different concentrations (0.2%, 0.4%, 0.6%, 0.8%, and 1.0%) of SA were used. After mutagenesis, the seeds were washed to remove any chemical residue. One hundred seeds of each concentration and control for both species were sown in five replicates in 12-inch earthen pots containing 7.5 kg of soil in the Net House of the Department of Botany, Aligarh Muslim University, during the rabi season of 2019–20 to raise the M_1_ generation. The experimental site is characterized by a semi-arid and sub-tropical climate, featuring hot and dry summers and cold winters. The region experiences an average rainfall of 847.30 mm, while the summer and winter average temperatures are 35°C and 15°C, respectively. The soil in Aligarh is sandy loam and has an alkaline nature. Seed germination was monitored by observing the emergence of cotyledons. The experiment aimed to examine the impact of the chemical mutagen on growth parameters in the M_1_ generation, and the results were carefully screened and analyzed. The seed germination, plant survival, and pollen fertility percentage of both varieties were assessed in the M_1_ generation.

## Chlorophyll and carotenoid content

The formulas for calculating chlorophyll a (Chl a) and chlorophyll b (Chl b) content are as follows:

$$\text{Chlorophyll }a\;\left(\text{mg}/g\right)=\left[12.7\;\left({\text{OD}}_{663}\right)+2.69\;\left({\text{OD}}_{645}\right)\right]\frac{V}{1000\times w\;},$$

$$\text{Chlorophyll }b\;\left(\text{mg}/g\right)=\left[22.9\;\left({\text{OD}}_{645}\right)+4.68\;\left({\text{OD}}_{663}\right)\right]\frac{V}{1000\times w\;}.$$

The total chlorophyll content of leaves can be estimated by Arnon’s method and was calculated using the formula given below:

$$\text{Total chlorophyll content }\left(\text{mg}/g\right)=\left[20.2\;\left({\text{OD}}_{645}\right)+8.02\;\left({\text{OD}}_{663}\right)\right]\frac{V}{1000\times w\;},$$

$$\text{Carotenoid content }\left(\text{mg}/g\right)=\left[7.6\;\left({\text{OD}}_{480}\right)\;–\;1.49\;\left({\text{OD}}_{510}\right)\right]\frac{V}{1000\times d\times w}.$$

OD_645_, OD_663_, OD_480_, and OD_510_ are the optical densities of 645 nm, 663 nm, 480 nm, and 510 nm, respectively; V is the volume of an extract; W is the mass of leaf tissues; d is the length of light path (1.4 cm).

## Proline content

Proline content was extracted from fresh leaves using 3% aqueous sulphosalicylic acid, and the extract was filtered. In a glass test tube, 2 mL of the filtrate was mixed with 2 mL of glacial acetic acid and 2 mL of acid ninhydrin. The test tube was then heated in a boiling water bath for 1 h and later placed in an ice bath. To the reaction mixture, 4 mL of toluene was added and stirred for 20–30 s. The optical density of the resulting color was measured at 520 nm to determine the proline content (Bates et al., 1973).

## Quantitative traits

Data on quantitative traits were collected from a sample of 15 randomly selected plants from each replication and presented as the mean. Plant height is determined by measuring the distance from the base of the plant to the highest point or apex of the plant. The number of fertile branches per plant was assessed by counting the branches at the maturity stage. Pods per plant were determined by counting the total number of pods collected from successive harvests. Seeds per pod were calculated by counting the seeds in randomly selected pods from the 15 plants. Seed weight was determined by weighing 1,000 seeds collected from a random selection of 15 plants. The total plant yield was assessed by weighing all the seeds collected from the 15 randomly selected plants.

## Cytological analysis

To conduct cytological studies, young flower buds were collected individually from each treatment group and immersed in Carnoy’s fluid (a mixture of one part glacial acetic acid, three parts chloroform, and six parts ethyl alcohol) for 24 h to fix the tissues. After fixation, the flower buds were transferred to 70% alcohol for further preparation. Anthers from the flower buds were then smeared in 1% acetocarmine, and various stages of microsporogenesis were observed using a compound microscope to study their behavior. Photographic documentation was done using temporary preparations.

## Stomatal investigation of leaves and micromorphological analysis of seeds

For this study, leaf variants and seed variants were subjected to scanning electron microscopy (SEM) to investigate stomatal characteristics and seed micromorphology, respectively. The samples were fixed using 2.5% glutaraldehyde and dehydrated with an alcohol series (30%, 50%, 70%, 90%, and 100%) for 15–20 min. Subsequently, they were mounted on aluminum stubs and coated with gold for 4 min in a sputter coater. The SEM analysis was carried out using a JEOL JSM-6510LV scanning electron microscope at the University Sophisticated Instrumentation Facility (USIF) at Aligarh Muslim University, Aligarh. Micrographs were taken at 33× and 3,000× magnification at 15 kV to observe the stomatal aperture size in leaves, seed size, and the plant’s surface features.

## Statistical analysis

The data obtained from the study were subjected to statistical analysis using R software. To determine the differences between treatments, an analysis of variance (one-way ANOVA) was conducted, and the Duncan multiple range test (*p* ≤ 0.05) was used to compare the means of different treatments. The significant differences between groups were analyzed by two-way analysis of variance (two-way ANOVA, *p* < 0.05) using GraphPad Prism 10. Additionally, Pearson correlation analysis and a heat map were generated using R software version 4.2.2 to examine the relationships between different variables and present the data in a graphical format.

## Results

### Effect of sodium azide on germination, plant survival, and pollen fertility

**FIGURE 1:**
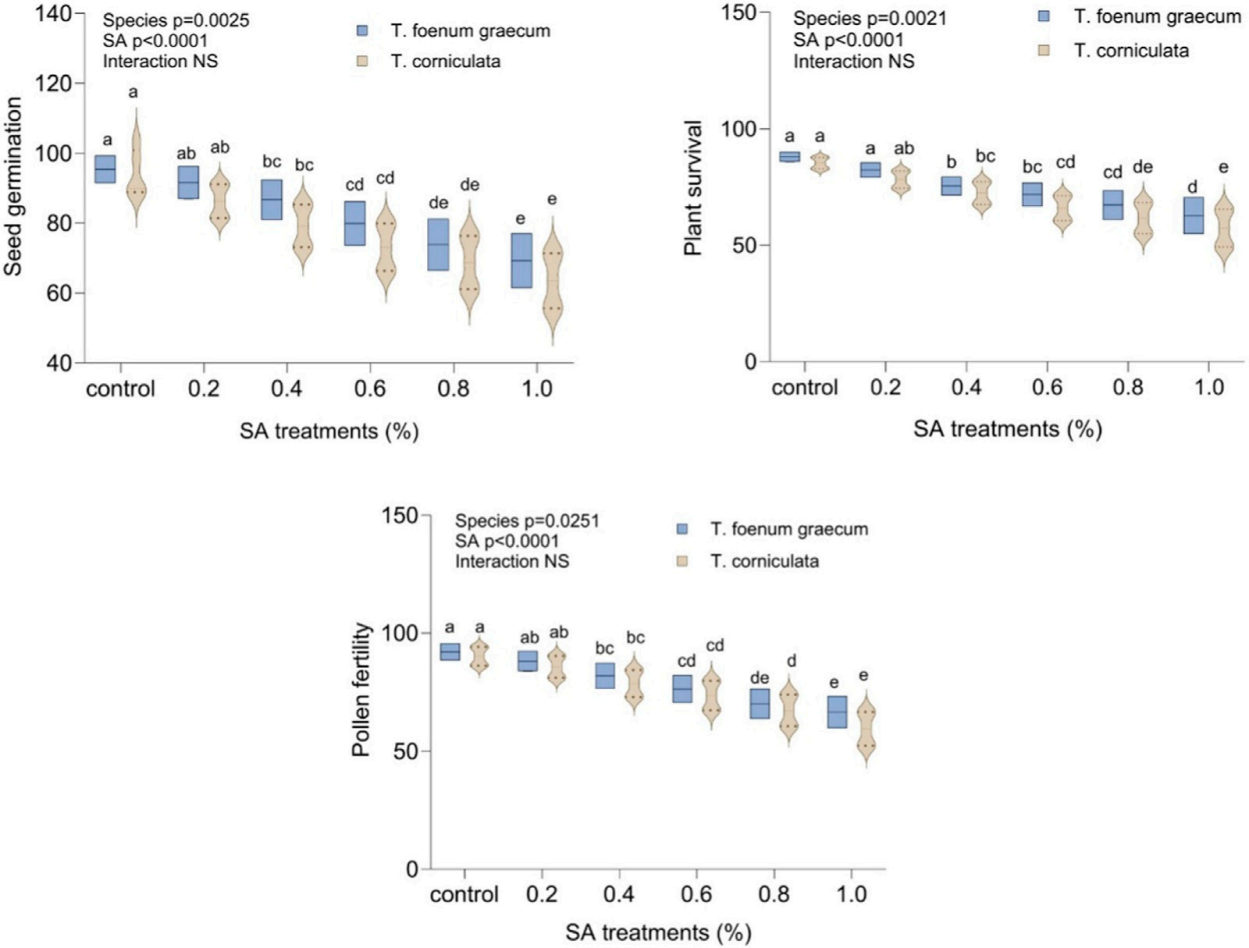
Effect of SA on seed germination, plant survival, and pollen fertility of *Trigonella foenum-graecum* and *Trigonella corniculata*. Data are presented as mean ± SE of five replicates (*n* = 5) of each treatment. Error bars with different lowercase letters are significant at the 5% level of significance, tested by the Duncan multiple range test (DMRT). Significant differences between groups were analyzed by two-way analysis of variance (Two-way ANOVA, *p* < 0.05). NS, not significant.

Figure 1 presents the impact of varying concentrations of SA on seed germination, plant survival, and pollen fertility in fenugreek species. SA application resulted in a decrease in all these parameters compared to untreated plants. In the control group, seed germination was 95.0 for *T. foenum-graecum* and 93.0 for *T. corniculata.* With SA treatment, germination significantly decreased (*p* < 0.05) to a range of 91.0 to 69.0 in desi methi and 86.0 to 63.0 in kasuri methi. The reduction was 27.4% in desi methi and 32.3% in kasuri methi in the 1.0% treatment groups. Plant survival also declined significantly (*p* < 0.05) with higher SA concentration, particularly in kasuri methi (32.8%) compared to desi methi (28.7%). Additionally, pollen fertility showed a decrease of 27.5% in desi methi and 33.9% in kasuri methi due to SA treatment. Kasuri methi displayed higher sensitivity to mutagenic treatments than desi methi.

### Effect of sodium azide on chlorophyll, carotenoids, and proline content

**FIGURE 2:**
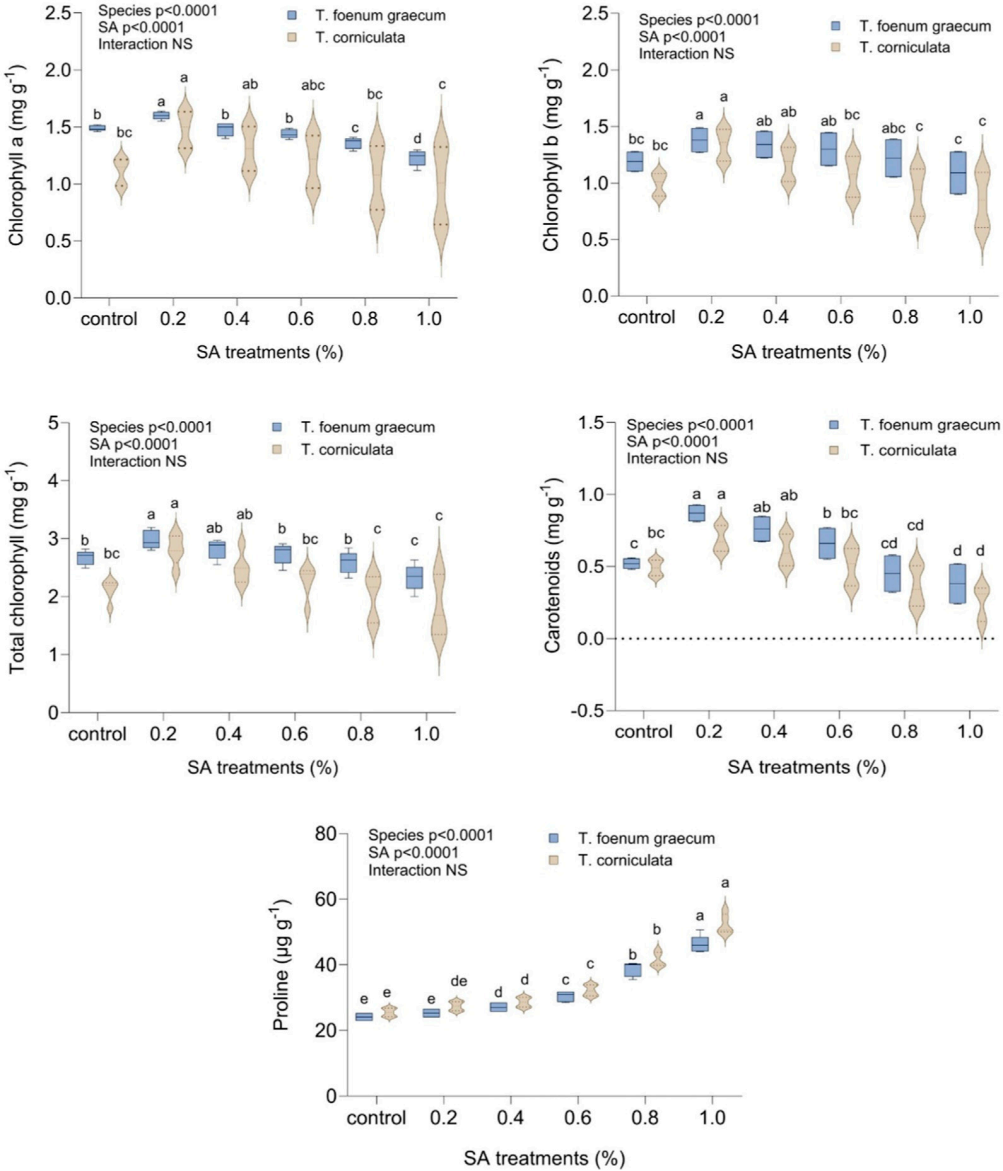
Effect of SA on chlorophyll a, chlorophyll b, total chlorophyll, carotenoids, and proline content of *Trigonella foenum-graecum* and *Trigonella corniculata.* Data are presented as mean ± SE of five replicates (*n* = 5) of each treatment. Error bars with different lowercase letters are significant at the 5% level of significance, tested by the Duncan multiple range test (DMRT). Significant differences between groups were analyzed by two-way analysis of variance (two-way ANOVA, *p* < 0.05). NS, not significant.

Figure 2 displays the response of fenugreek leaves’ photosynthetic pigments to different concentrations of SA treatment. The results demonstrated a significant (*p* < 0.05) increase in all photosynthetic pigment contents (chlorophyll a, chlorophyll b, carotenoids, and total pigments) with increasing SA concentrations, up to 0.2%. In the control group, chlorophyll content was 2.67 mg g^−1^ and 2.11 mg g^−1^, while carotenoids were 0.52 mg g^−1^ and 0.49 mg g^−1^ in desi methi and kasuri methi, respectively. At 0.2% SA, chlorophyll and carotenoids increased by 11.6% and 67.3% in desi methi and 33.2% and 40.8% in kasuri methi, respectively. However, doses higher than 0.2% SA doses led to a significant reduction in chlorophyll and carotenoid content in both species. Proline content continuously increased with rising SA concentrations compared to the control plants. Higher SA treatments resulted in a notable increase in proline content, increased by 92.1% in kasuri methi and 85% desi methi at 1.0% SA. The treatment effect was significant for the studied traits, but the interaction effect between the species and the treatment was not significant in these parameters.

### Effect of sodium azide on yield and its attributing traits

In this study, we recorded observations on several quantitative traits viz., plant height (cm), number of branches per plant, number of pods per plant, number of seeds per pod, 1,000 seed weight (g), total yield per plant (g), number of clusters per plant and number of pods per cluster in the M_1_ generation of two plant species. By observing and analyzing these quantitative traits in the M_1_ generation of both plant species, the researchers can gain valuable insights into the genetic variations, heritability, and potential effects of any treatments or environmental factors on the growth, development, and yield-related characteristics of the plants.

**FIGURE 3:**
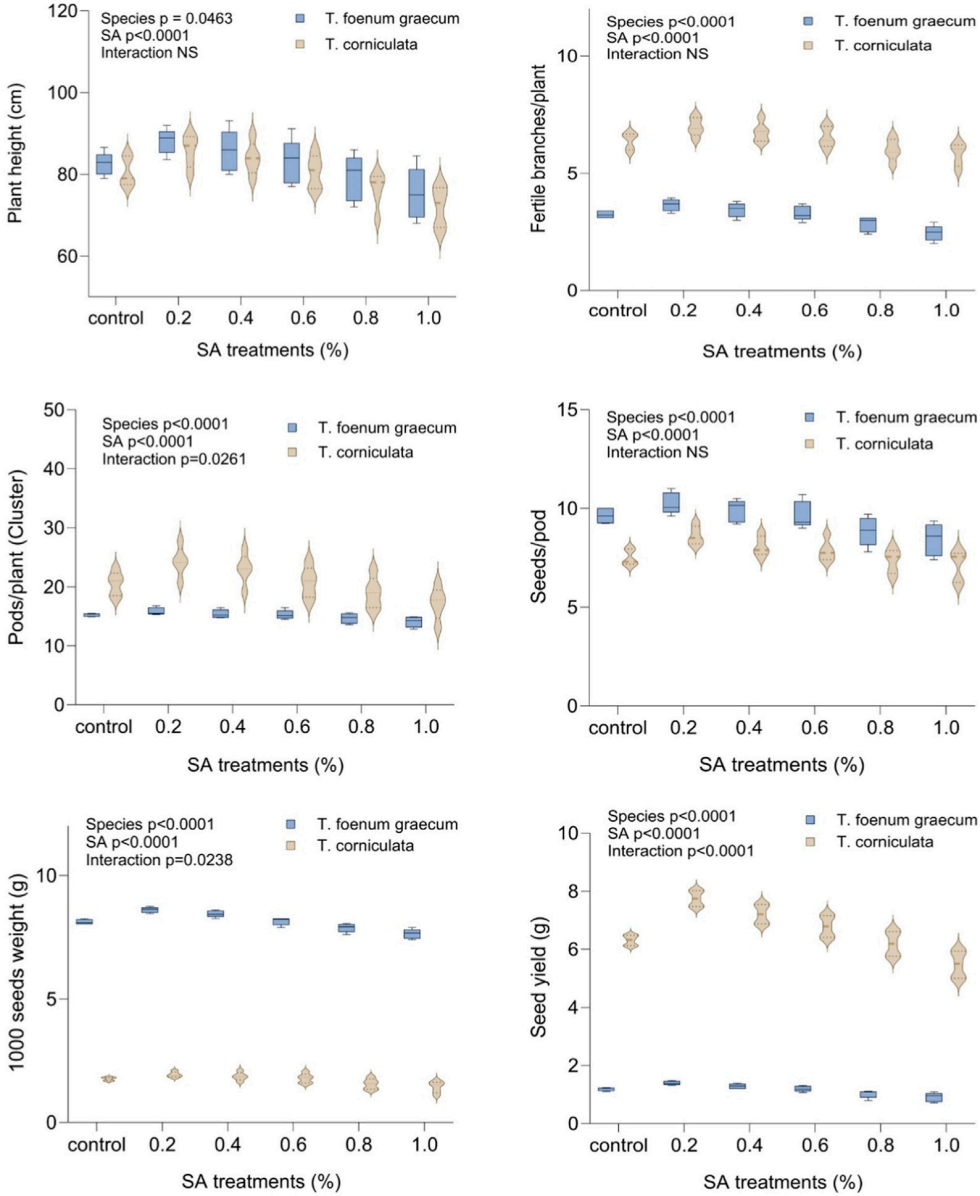
Effect of SA on plant height, fertile branches/plant, pods/plant (cluster), seeds/pod, 1,000-seed weight, and seed yield of *Trigonella foenum-graecum* and *Trigonella corniculata.* Data are presented as mean ± SE of five replicates (*n* = 5) of each treatment. Significant differences between groups were analyzed by two-way analysis of variance (two-way ANOVA, *p* < 0.05). NS, not significant.

In the control group, the mean plant height was 82.50 cm in desi methi and 80.60 cm in kasuri methi. When treated with 0.2% SA, both *T. foenum-graecum* and *T. corniculata* showed the highest plant height values compared to the control. At 0.2% SA, the plant height increased by 6.78% in desi methi and 6.45% in kasuri methi. Conversely, higher concentrations of SA led to significant (*p* < 0.05) reductions in plant height, with a decrease of 8.7% in desi methi and 10.5% in kasuri methi compared to the control. The number of fertile branches per plant in both species significantly increased (*p* < 0.05) at 0.2% SA. The average number of branches per plant was 3.25 and 6.38 in the control for desi methi and kasuri methi, respectively. At 0.2% SA, there was a marked enhancement of 12.3% in desi methi and of 9.4% in kasuri methi. Regarding the number of pods per plant, *T. foenum-graecum* showed a slight increase of 3.9% in response to lower-concentration mutagenic treatments compared to the control group. In contrast, for *T. corniculata*, the mean value of the number of pods per cluster significantly (*p* < 0.05) increased by 18.3% at 0.2% SA treatment, but with higher SA concentrations, these values gradually decreased by 16.1%. The lower concentration SA treatment (0.2%) increased the average number of seeds per pod by 6.5% in desi methi and 14.7% in kasuri methi, as well as the 1,000-seed weight by 6% in desi methi and 9.6% in kasuri methi. The total yield per plant differed between the two species. With the 0.2% SA treatment, the yield per plant significantly increased (*p* < 0.05) to 1.40 g in desi methi and 7.75 g in kasuri methi. This represented a yield increase of 17.6% in desi methi and 22.8% in kasuri methi. There was a highly significant interaction effect between the species and treatments observed for the number of pods per plant (cluster), 1000-seed weight, and total seed yield per plant (Figure 3). The data collected from these observations can provide important information for understanding the phenotypic characteristics and performance of the plants, which may have implications for further breeding and agricultural applications.

### Meiotic aberrations

**FIGURE 4:**
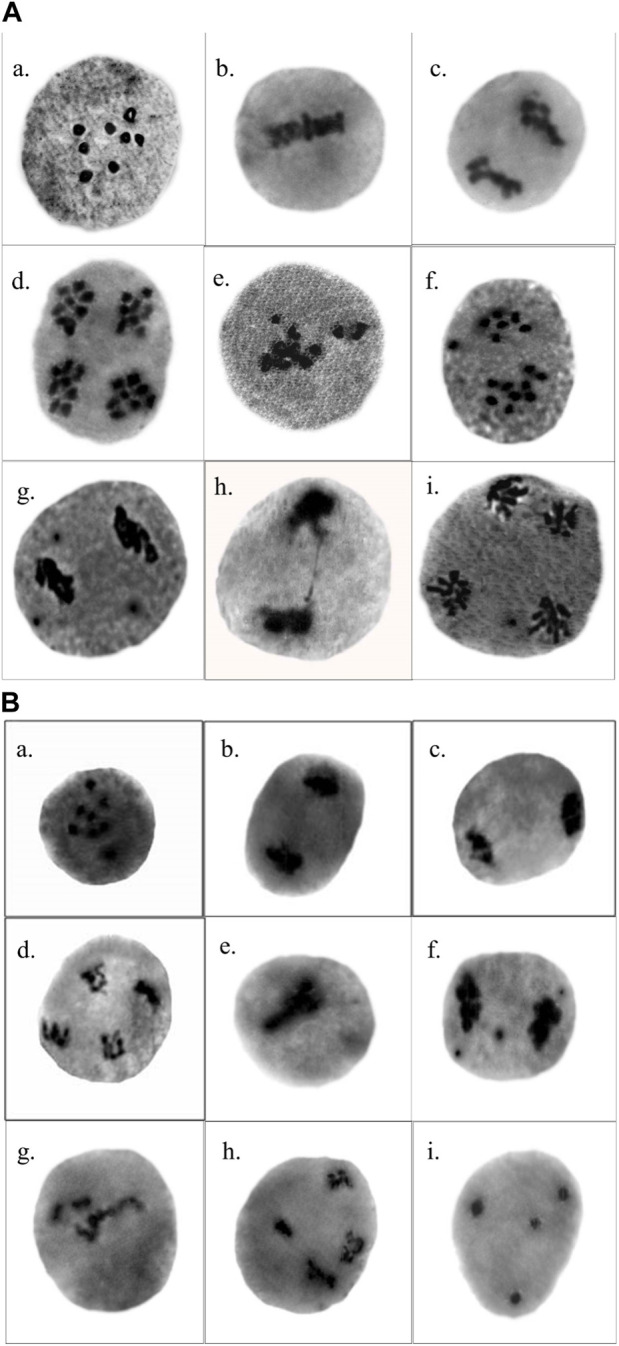
Chromosomal abnormalities induced by SA in **(A)** *Trigonella foenum-graecum* **(a)** prophase I—diakinesis, **(b)** metaphase I, **(c)** anaphase I, **(d)** telophase II, **(e)** disturbed metaphase I, **(f)** anaphase I—unequal separation and one laggard, **(g)** sticky anaphase I with three laggards, **(h)** chromatin bridge at anaphase I, and **(i)** laggard at anaphase II; and **(B)** *Trigonella corniculata* **(a)** prophase I—diakinesis, **(b)** anaphase I, **(c)** telophase I, **(d)** telophase II, **(e)** stickiness at metaphase I, **(f)** anaphase I with three laggards, **(g)** stray chromosomes, **(h)** unsynchronized anaphase II, and **(i)** disturbed polarity.

In the control plants of *T. foenum-graecum* and *T. corniculata*, normal meiotic division was observed with eight perfect bivalents (2n = 16) at metaphase, anaphase, and telophase. However, after mutagen treatment, the mutagenized population displayed various chromosomal aberrations in the pollen mother cells. In *T. foenum-graecum*, the selected variants exhibited abnormalities such as disturbed metaphase I, unequal separation of chromosomes resulting in configurations like (9 + 6) with one laggard at anaphase I, sticky anaphase I with three laggards, chromatin bridge at anaphase I, and laggard at anaphase II (Figure 4Aa-i). Conversely, in *T. corniculata*, the mutagenized plants exhibited chromosomal abnormalities, including stickiness, laggards, stray chromosomes, bridges, unequal separation of chromosomes, and disturbed polarity (Figure 4Ba-i). The frequency of these chromosomal aberrations in the mutagenized plants varied with the concentration of the mutagen. The highest mutational frequency in chromosomal structure was observed at a 1.0% SA concentration in *T. foenum-graecum* and *T. corniculata*. Moreover, the chromosomal abnormalities showed a dose-dependent relationship, increasing as the mutagen concentration increased. The total percentage of abnormal pollen mother cells (PMCs) ranged from 4.41% to 23.26% in *T. foenum-graecum* and 5.56%–26.09% in *T. corniculata* (Table 1). These findings demonstrate that the mutagen treatment induced significant chromosomal aberrations in both *T. foenum-graecum* and *T. corniculata*, and the extent of these abnormalities was linked to the concentration of the mutagen used.

**TABLE 1:** Frequency of chromosomal abnormalities induced by sodium azide in *Trigonella foenum-graecum* and *Trigonella corniculata*.

Treatments (SA %)		*Trigonella foenum-graecum*						*Trigonella corniculata*					
		Control	0.2%	0.4%	0.6%	0.8%	1.0%	Control	0.2%	0.4%	0.6%	0.8%	1.0%
Total no. of pollen mother cells (PMCs) observed		279	272	258	279	264	288	275	288	281	291	270	276
Prophase I (diakinesis)	Univalents	-	-	1	2	3	3	**-**	-	1	2	3	4
	Multivalents	-	-	1	1	2	4	**-**	1	2	2	3	4
	% of abnormal PMCs	-	0.00	0.78	1.08	1.89	2.43	**-**	0.35	1.07	1.37	2.22	2.90
Metaphase I/II	Univalents	-	-	1	1	2	4	**-**	1	2	2	3	5
	Multivalents	-	1	-	3	2	5	**-**	1	2	2	3	4
	Precocious movement	-	1	2	2	3	3	**-**	1	-	3	4	5
	Stray chromosome	-	1	-	2	4	4	**-**	-	1	2	2	3
	Stickiness	-	1	2	2	3	5	**-**	1	1	2	3	4
	% of abnormal PMCs	-	1.47	1.94	3.58	5.30	7.29	**-**	1.39	2.14	3.78	5.56	7.61
Anaphase I/II	Laggards	-	1	1	3	3	3	**-**	1	2	3	4	5
	Bridges	-	1	2	2	3	4	**-**	1	2	2	2	4
	Unequal separation	-	1	1	2	2	4	**-**	1	1	2	3	3
	% of abnormal PMCs	-	1.10	1.55	2.51	3.03	3.82	**-**	1.04	1.78	2.41	3.33	4.35
Telophase I/II	Laggards	-	1	1	3	1	1	**-**	1	1	3	4	5
	Bridges	-	1	-	1	-	1	**-**	1	2	2	3	4
	Unequal separation	-	-	1	2	1	-	**-**	2	2	2	3	5
	Micronucleate	-	1	1	3	1	1	**-**	1	2	3	4	4
	Multinucleate	-	-	1	3	1	-	**-**	-	1	3	2	5
	Disturbed polarity	-	1	2	2	2	1	**-**	1	1	2	3	4
	Cytomixis	-	1	1	2	1	1	**-**	2	1	3	2	4
	% of abnormal PMCs	-	1.84	2.71	5.73	7.58	9.72	**-**	2.78	3.56	6.19	7.78	11.23
Total no. of abnormal PMCs observed		-	12	18	36	47	67	**-**	16	24	40	51	72
Total % of abnormal PMCs observed		-	4.41	6.98	12.90	17.80	23.26	**-**	5.56	8.54	13.75	18.89	26.09

### Stomatal study of leaves and micromorphology of seeds

**FIGURE 5:**
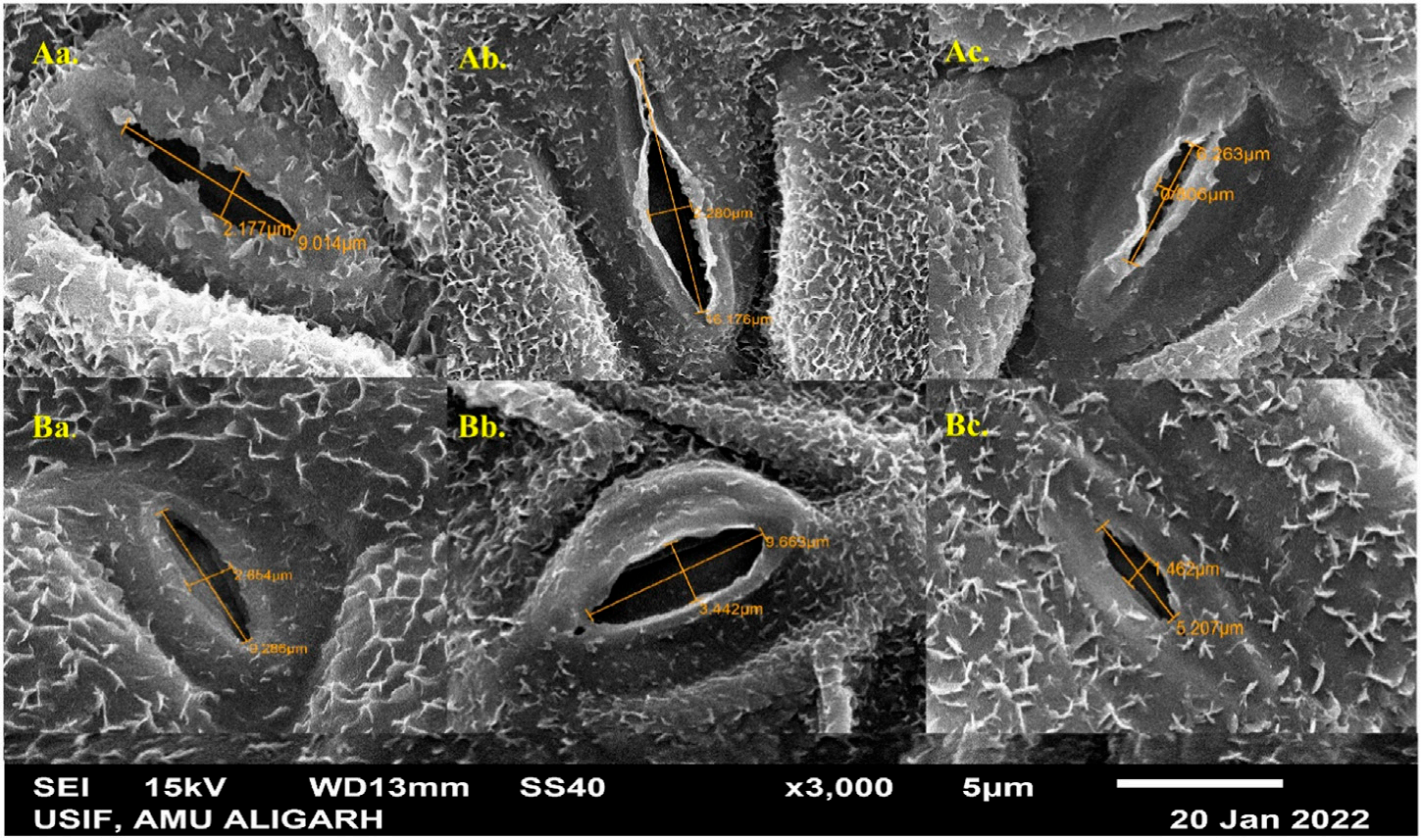
SEM microphotographs of stomata of SA-treated **(A)** *Trigonella foenum-graecum* and **(B)** *Trigonella corniculata.*

The study examined the stomatal characteristics of leaves and the micromorphology of seeds in both species. Structural variations in the guard cells and leaf stomata were observed in the selected variants. SA had more pronounced toxic effects on *T. corniculata* stomata than *T. foenum-graecum*. At higher concentrations of the mutagen, the shape and size of the stomatal aperture appeared to be distorted. When comparing with the control plants (Figures 5Aa, Ba), the maximum stomatal lengths were 16.176 μm and 9.663 μm, observed at the 0.1% SA concentration in desi methi (Figure 5Ab) and kasuri methi (Figure 5Bb), respectively. This represented an increase of 65.6% in desi methi and 14.1% in kasuri methi. Additionally, the width of the stomata increased at lower concentrations compared to control plants. Increments of 2.2% and 30.1% in desi methi and kasuri methi, respectively, were observed at 0.2% SA. However, at the 1.0% SA concentration, the minimum widths of the stomata were 0.806 μm and 1.462 μm in desi methi (Figure 5Ac) and kasuri methi (Figure 5Bc), respectively, which are reductions of 36.8% and 39.6% compared to the control.

**FIGURE 6:**
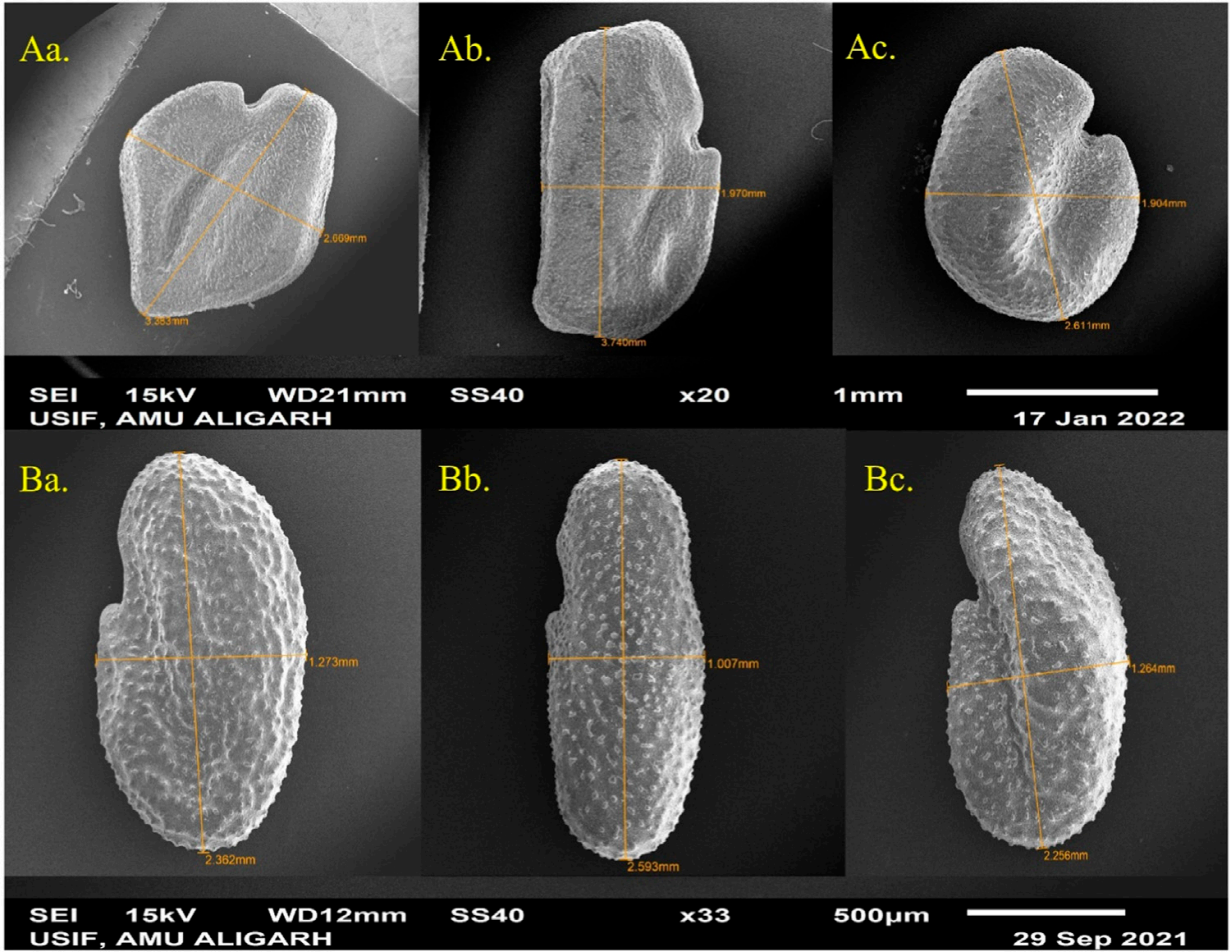
SEM microphotographs of SA-treated seed of **(A)** *Trigonella foenum-graecum* and **(B)** *Trigonella corniculata.*

**FIGURE 7:**
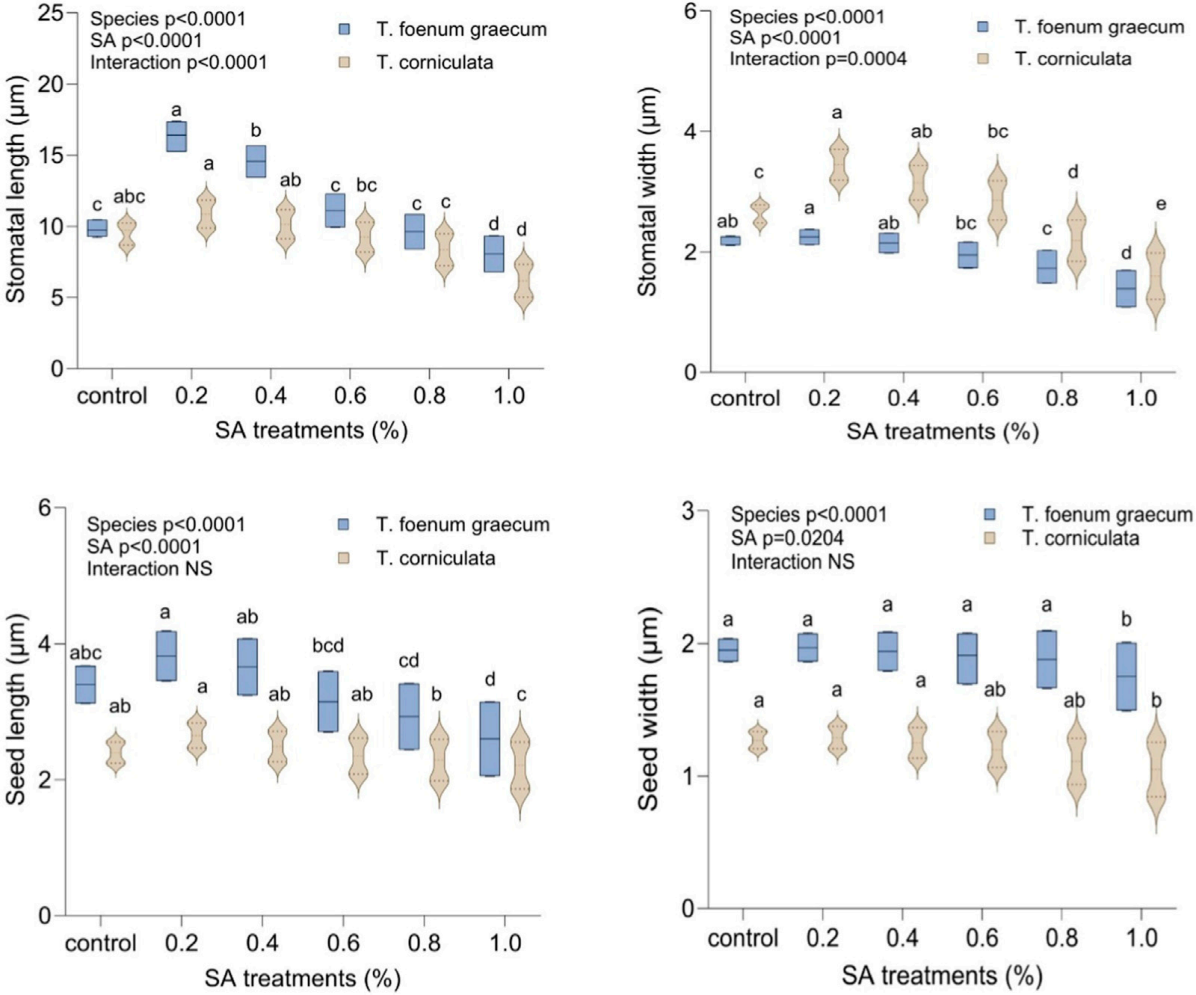
Effect of SA on stomatal length, stomatal width, seed length, and seed width of *Trigonella foenum-graecum* and *Trigonella corniculata.* Data are presented as mean ± SE of five replicates (*n* = 5) of each treatment. Error bars with different lowercase letters are significant at the 5% level of significance, tested by the Duncan multiple range test (DMRT). Significant differences between groups were analyzed by two-way analysis of variance (two-way ANOVA, *p* < 0.05). NS, not significant.

In terms of seed characteristics (Figure 6), SEM analysis revealed variations in seed shape and size in both species. The maximum seed size was observed at lower concentrations of the mutagen, measuring 3.740 μm × 1.970 μm for *T. foenum-graecum* (Figure 6Ab) and 2.593 μm × 1.007 μm for *T. corniculata* (Figure 6Bb). An enhancement of 12.3% and 10.4% in seed length of desi methi and kasuri methi at 0.2% SA, respectively, was observed, while seed width increased by 1% and 1.5% in desi methi and kasuri methi, respectively. For *T. foenum-graecum*, the control seeds were rhomboidal in shape with lateral and oblique grooves (Figure 6Aa). However, elongated (oblong) seeds with irregular shapes (Figure 6Ab) and small oval-shaped seeds (Figure 6Ac) were also detected. Conversely, the control seeds of *T. corniculata* were round in shape (Figure 6Ba), and elongated seeds (Figure 6Bb) and distorted shapes with one side compressed (Figure 6Bc) were observed. A highly significant interaction effect between the species and treatments was observed for the stomatal aperture length and stomatal aperture width (Figure 7). Overall, the study indicates that SA treatment led to significant changes in the stomatal characteristics of both species and influenced the micromorphology of their seeds, with some effects being more pronounced at higher concentrations of the mutagen.

### Pearson correlation analysis

**FIGURE 8:**
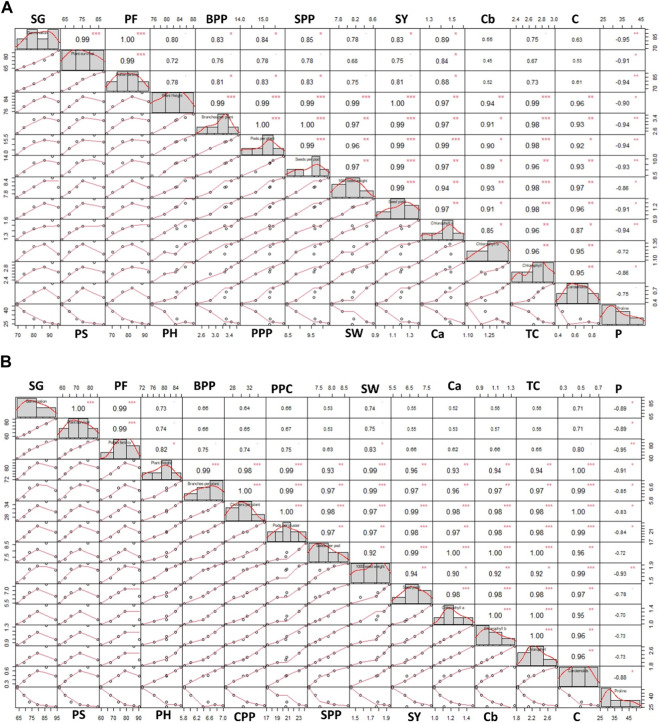
Pearson correlation coefficient between the parameters of **(A)** *Trigonella foenum-graecum* and **(B)** *Trigonella corniculata.* Significant differences are indicated as *p ≤ 0.05, **p ≤ 0.01, and ***p ≤ 0.001. SG, seed germination; PS, plant survival; PF, pollen fertility; PH, plant height; BPP, branches per plant; PPP, pods per plant; CPP, cluster per plant; SPP, seeds per pod; SW, seed weight; SY, seed yield; Ca, chlorophyll a; Cb, chlorophyll b; TC, total chlorophyll; C, carotenoids; P, proline.

Pearson correlation analysis was conducted to investigate the relationships among all the studied parameters of both *Trigonella* species. In *T*. *foenum-graecum*, most of the parameters showed a positive correlation, as depicted in Figure 8A. Specifically, high positive correlations (r = 1.00) were observed between germination and pollen fertility, plant height, and seed yield, as well as between branches per plant and pods per plant, indicating that these traits tend to change together consistently. Similarly, in *T*. *corniculata*, high positive correlations (r = 1.00) were observed between germination and plant survival, branches per plant and clusters per plant, and clusters per plant and pods per cluster, as shown in Figure 8B. These findings suggest that these traits are closely related and tend to vary in a synchronized manner. Additionally, other parameters in both species also exhibited positive correlations, indicating some degree of association between various traits.

### Heatmap analysis

**FIGURE 9:**
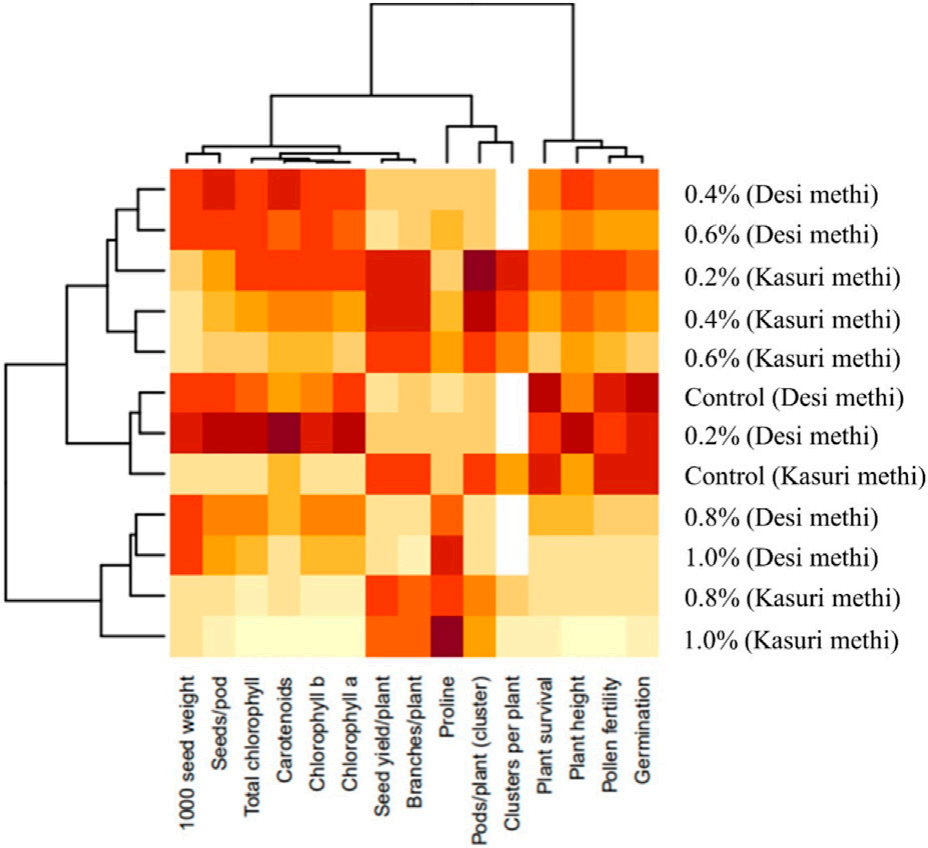
Heatmap analysis of all the parameters of SA-treated desi methi and kasuri methi.

A heatmap is a visual representation of data that uses colors to convey information about the values of different parameters. In the context of this study, a heatmap was used to evaluate the clustering pattern of the studied parameters in both *Trigonella* species. Each row in the heatmap (Figure 9) represents a specific parameter, and each column represents a different variant or treatment. The colors in the heatmap indicate the relative abundance or content of each parameter. Parameters with similar abundance or content are grouped together, making it easier to identify patterns and relationships among the variables. From the heatmap analysis, it was observed that “branches per plant,” “pods per plant (cluster),” and “seed yield” clustered together. This suggests that these parameters may be related or have similar trends in their responses to different variants or treatments. The clustering of these parameters indicates that they might share common characteristics or might be influenced by similar factors. On the other hand, the parameters “proline content,” “seeds per pod,” and “1,000 seed weight” did not cluster together under any specific group. This suggests that these parameters may have unique characteristics and may respond differently to the studied variants or treatments. Overall, the heatmap provides a useful classification and overview of the studied parameters based on their content or abundance.

## Discussion

The focus of the current discussion is on the effects of a specific chemical mutagen, sodium azide (SA), on various biological parameters in the M_1_ generation of two *Trigonella* species, *T. foenum-graecum* and *T. corniculata*. The overall goal is to assess the mutagenic effect of SA on these *Trigonella* species and explore the potential for creating novel genotypes with desirable agricultural traits.

Seed germination is a vital process that signifies the restoration of active metabolism and visible growth in plants. In this study, the increase in mutagenic doses led to a reduction in seed germination, plant survival, and pollen fertility. Similar effects have been reported in various plant species, including *Trigonella foenum-graecum* (Hassan et al., 2018; Naaz et al., 2020), *Lens culinaris* (Sharma et al., 2022), *Linum usitatissimum* (Jahan et al., 2020), *Nigella sativa* (Tantray et al., 2017), *Capsicum annum* (Aslam et al., 2017), *Psoralea* (Bhat et al., 2015), and mungbean (Veni et al., 2017). However, the degree of reduction varied between the two *Trigonella* species studied. The inhibition of seed germination can be attributed to disruptions in endogenous growth regulators crucial for the germination process, resulting from imbalances between the promoters and inhibitors involved in seed germination (Aman, 1986). Thus, high concentrations of growth inhibitors in pericarps and seed coats can hinder embryo germination (Bhat et al., 2015). Various factors, including cytogenetic and physiological disturbances, imbalances in metabolic activity, and disruptions in the interplay of growth regulators, may contribute to the reduction in plant survival following mutagenic treatments (Al-Qurainy, 2009; Girija et al., 2013). Notably, *T. corniculata* exhibited higher sensitivity to mutagenic treatments than *T. foenum-graecum* (Figure 1).

The mutagen-treated population exhibited significant variation in chlorophyll content and carotenoids, indicating potential alterations in photosynthetic activity, consistent with previous research on *Trigonella* (Hassan et al., 2018; Naaz et al., 2020) and *Capsicum annum* (Hasan et al., 2020). The rise in total chlorophyll and carotenoid levels in mutants was primarily due to elevated levels of chlorophyll a and β-carotene, as observed in earlier studies (Tomlekova et al., 2009). The increased activity of chlorophyllase, an enzyme responsible for chlorophyll degradation, may contribute to the reduction in chlorophyll content (Reddy and Vora, 1986). Carotenoids play a crucial role in photosynthesis and also act as antioxidants, protecting chlorophyll from photodamage. Additionally, carotenoids possess antioxidant properties that reduce oxidative stress in the body (Fiedor and Burda, 2014). Proline, an important osmotic-reluctance substance in plants, plays a vital role in controlling cell function, protecting cell membranes, and providing stability to biological macromolecules (Nadar and Rathod, 2020). The elevated proline content in mutagen-treated plants with increased SA concentration underscores enhanced osmotolerance, consistent with findings in *Dracaena sanderiana* (Junaid et al., 2008), *Coriandrum sativum* (Kumar and Pandey, 2019), and *C. annum* (Hasan et al., 2022). This highlights proline’s crucial role in stress tolerance and osmoregulation, as observed in various plant species (Figure 2).

In this study, a significant increase in quantitative traits was observed at lower and intermediate concentrations of the mutagen compared to the control group. These traits included plant height, pods bearing branches per plant, number of pods per plant, number of seeds per pod, and overall yield. The mutagenic treatment induced variations in these traits, resulting in enhanced growth and productivity of the plants. Here, it was observed that the average plant height significantly increased compared to the control at lower and intermediate concentrations of mutagens. However, as the mutagenic treatments increased, the plant height generally decreased. Previous studies have also reported a reduction in plant height in various plant species, including *Capsicum* (Hasan et al., 2022), *Trigonella* (Naaz et al., 2023), *Psoralea corylifolia* (Bhat et al., 2015), *Glycine max* (Ahire and Auti, 2015), and *Cajanus cajan* (Ariraman et al., 2018). The decrease in plant height could be attributed to damage to meristematic tissues, metabolic disturbances, and physiological variations.

A notable increase in the mean number of branches was observed at lower concentrations of mutagens, consistent with previous findings in fenugreek, urdbean, and lentil (Naaz et al., 2020; Goyal et al., 2022; Sharma et al., 2022). This increase in fertile branches per plant may be associated with reduced synthesis of strigolactones, influencing branching patterns. Additionally, studies have reported a significant increase in the number of pods per plant in crops like *Nigella sativa* (Amin et al., 2019) and *Sesamum indicum* (Pradhan and Paul, 2019), positively correlating with the total yield per plant. The higher number of pods and seeds directly impacts the overall yield per plant. Here, higher mutagen doses had inhibitory effects on yield, while lower and moderate doses resulted in a significant increase in seed yield in the M_1_ generation. The decrease in yield parameters following mutagenic treatments has been observed across various plant species, including *Trigonella* (Choudhary et al., 2012), *Lens culinaris* (Laskar and Khan, 2017), *Capsicum annuum* (Aslam, 2015), *Linum* (Jahan et al., 2019), and *N. sativa* (Amin et al., 2019). This decrease in yield might be attributed to disruptions in meiosis, alterations in normal microspore and megaspore frequency, physiological disturbances, chromosomal damage, disturbed spindle formation, restricted pairing, prolonged DNA synthesis, and high pollen sterility (Figure 3).

Chromosomal studies examining meiotic behavior serve as reliable indicators for assessing the effectiveness of mutagens (Table 1). The presence of stray chromosomes at metaphase can be attributed to spindle dysfunction and clumping of chromosomes (Bhat et al., 2007). Additionally, the precocious separation of univalents and the appearance of bivalents as stray chromosomes may lead to unequal distribution of chromosomes or loss of complete bivalents during later stages (Khan et al., 2009). Chromosomal stickiness, caused by the depolymerization of nucleic acids or incomplete dissociation of nucleoproteins after exposure to mutagens, alters chromosomal organization. Bridges observed during anaphase, resulting from chiasma non-separation due to stickiness, may be caused by gene mutations or direct effects of mutagens on proteins, leading to malfunctioning of target proteins during chromosome separation (Kumar and Gupta, 2009). Laggards, observed in some instances, are attributed to abnormal spindle fibers failing to bind and transport respective chromosomes to the polar region during meiosis (Tarar and Dnyansagar, 1980). These findings align with previous research on various plant species such as *T. foenum-graecum* (Choudhary et al., 2012; Naaz et al., 2023), *C. annum* (Hasan et al., 2022), and *L. culinaris* (Sharma et al., 2022), emphasizing the significance of chromosomal studies in understanding the complex effects of mutagens on chromosome behavior and structure during meiosis (Figure 4).

Stomata are vital for plant survival, serving key roles in photosynthesis, transpiration, and overall crop quality and yield. In this study, an increase in stomatal length and width was observed, while the width of stomata decreased (Figure 5). Previous research (Outlaw, 1983) suggests that stomatal opening is facilitated by the accumulation of ions and solutes in guard cells, resulting in decreased water potential and subsequent water uptake from the apoplast (Willmer and Fricker, 1996). This process leads to an increase in the volume of guard cells and turgor pressure, widening the stomatal pore (Shimazaki et al., 2007). The observed increase in stomatal size is anticipated to enhance the transpiration rate, thereby potentially improving photosynthesis, promoting plant growth, and ultimately enhancing overall crop production. Similar findings regarding increased stomatal length and width have been reported in previous studies (Mallick et al., 2016). In addition to stomatal variations, differences in seed shape, dimension, and micromorphology were observed using SEM (Figure 6). Micromorphological variations in seed morphology, stigmas, and pollen grains were evident (Ullah and Kim, 2018), contributing further to the overall diversity and potential adaptability of the plant species. These findings underscore the importance of studying stomatal and seed micromorphology, providing valuable insights into the physiological and reproductive aspects of plants, which can significantly impact crop improvement and breeding programs (Figure 7).

The Pearson correlation analysis provides valuable insights into how different traits are interconnected in both *Trigonella* species, allowing us to understand potential patterns and relationships that may influence their growth, development, and overall performance (Figure 8). A heatmap is a visual representation of data that uses colors to convey information about the values of different parameters. It helps researchers to identify potential relationships, trends, and similarities among the parameters, thereby offering valuable insights into the data and aiding in the interpretation of results. The visualization provided by the heatmap allows for a quick understanding of the data’s clustering patterns and can guide further analysis and investigations in the study of both *Trigonella* species (Figure 9).

The availability of ample genetic variability through mutagenesis provides breeders with a broader range of options for selecting desirable traits and ultimately improving crop performance and yield. The findings of this study demonstrate the potential of mutagenesis as an effective tool for generating genetic variability and facilitating trait improvement in crops.

## Conclusion

The study suggests that exposing fenugreek, a medicinal plant, to 0.2% and 0.4% concentrations of sodium azide can expand its genetic makeup, leading to noticeable changes in plant characteristics. *Trigonella corniculata* was found to be more susceptible to mutagenic treatments than *Trigonella foenum-graecum*, indicating species-specific responses to mutagenic agents. The preferred mutagenic concentration demonstrated high potency, resulting in significant phenotypic diversity within the treated population, which is advantageous for breeding programs. The higher the phenotypic diversity, the more potential there is to select plants with desirable traits for further cultivation and development. The observed positive correlation between studied parameters indicates promising prospects for developing high-yielding fenugreek lines. However, further evaluation over subsequent generations is necessary before integrating identified variants into breeding programs.

## Data Availability

The original contributions presented in the study are included in the article/Supplementary material; further inquiries can be directed to the corresponding author.
